# Phase-Specific Evaluation of Sciatic Nerve Regeneration in Preclinical Studies: A Review of Functional Assessment, Emerging Therapies, and Translational Value

**DOI:** 10.3390/ijms27010419

**Published:** 2025-12-31

**Authors:** Denisa Mădălina Viezuină, Irina (Mușa) Burlacu, Andrei Greșiță, Irina-Mihaela Matache, Elena-Anca Târtea, Mădălina Iuliana Mușat, Manuel-Ovidiu Amzoiu, Bogdan Cătălin, Veronica Sfredel, Smaranda Ioana Mitran

**Affiliations:** 1 U.M.F. Doctoral School Craiova, University of Medicine and Pharmacy of Craiova, 200349 Craiova, Romania; 2 Department of Physiology, University of Medicine and Pharmacy of Craiova, 2-4 Petru Rares Str., 200349 Craiova, Romania; bogdan.catalin@umfcv.ro (B.C.); veronica.sfredel@umfcv.ro (V.S.); smaranda.mitran@umfcv.ro (S.I.M.); 3 Experimental Research Centre for Normal and Pathological Aging, University of Medicine and Pharmacy of Craiova, 200349 Craiova, Romania; madalina.musat@umfcv.ro; 4 Faculty of Medicine, Carol Davila University of Medicine and Pharmacy, 050474 Bucharest, Romania; 5 Department of Neurology, University of Medicine and Pharmacy Craiova, 200349 Craiova, Romania; anca.tartea@umfcv.ro; 6 Department of Scientific Research Methodology, University of Medicine and Pharmacy of Craiova, 2-4 Petru Rares Str., 200349 Craiova, Romania; 7 Faculty of Pharmacy, University of Medicine and Pharmacy of Craiova, 200638 Craiova, Romania; manuel.amzoiu@umfcv.ro

**Keywords:** sciatic nerve, sciatic functional index, beam walk test, rotarod test, nerve regeneration

## Abstract

Peripheral nerve injuries, particularly those involving the sciatic nerve, remain a major clinical challenge due to incomplete functional recovery and the limited translation of preclinical advances into effective therapies. This review synthesizes current evidence on the phase-specific evaluation of sciatic nerve regeneration in preclinical models, integrating behavioral, sensory, electrophysiological, and morphological approaches across the acute, subacute (Wallerian degeneration), early regenerative, and late regenerative phases. By mapping functional readouts onto the underlying biological events of each phase, we highlight how tools such as the Sciatic Functional Index, Beam Walk test, Rotarod test, nerve conduction studies, and nociceptive assays provide complementary and often non-interchangeable information about motor, sensory, and neuromuscular recovery. We further examine emerging therapeutic strategies, including intraoperative electrical stimulation, immunomodulation, platelet-rich plasma, bioengineered scaffolds, conductive and piezoelectric conduits, exosome-based hydrogels, tacrolimus delivery systems, and small molecules, emphasizing the importance of aligning their mechanisms of action with the dynamic microenvironment of peripheral nerve repair. Despite substantial advancements in experimental models, an analysis of publication trends and registries reveals a persistent translational gap, with remarkably few clinical trials relative to the high volume of preclinical studies. To illustrate how mechanistic insights can be complemented by molecular-level characterization, we also present a targeted computational analysis of alpha-lipoic acid (ALA,) including frontier orbital energies, physicochemical descriptors, and docking interactions with IL-6, TGF-β, and a growth-factor receptor—performed solely for this molecule due to its documented structural availability and relevance. By presenting an integrated, phase-specific framework for functional assessment and therapeutic evaluation, this review underscores the need for standardized, biologically aligned methodologies to improve the rigor, comparability, and clinical relevance of future studies in sciatic nerve regeneration.

## 1. Introduction

The peripheral nervous system (PNS) is a vast and intricate network transmitting sensory and motor signals between the central nervous system and the rest of the body [1]. It takes part in the maintenance of homeostasis and the coordination of both voluntary and involuntary actions [2]. However, the extensive reach and exposure of the PNS render it very susceptible to various types of injuries from trauma, accidents, and different pathological conditions. Such injuries mainly cause significant sensory, motor, and autonomic dysfunctions, which can severely affect mobility and reduce quality of life [3].

Peripheral nerve injuries (PNIs) may arise through mechanisms such as ischemia, exposure to chemicals, mechanical trauma, and thermal damage [4]. Consequently, such injuries may cause damage to nerve fibers, disrupt communication between neurons and glial cells, and may also compromise the protective blood-nerve barrier [3,5].

**Figure 1:**
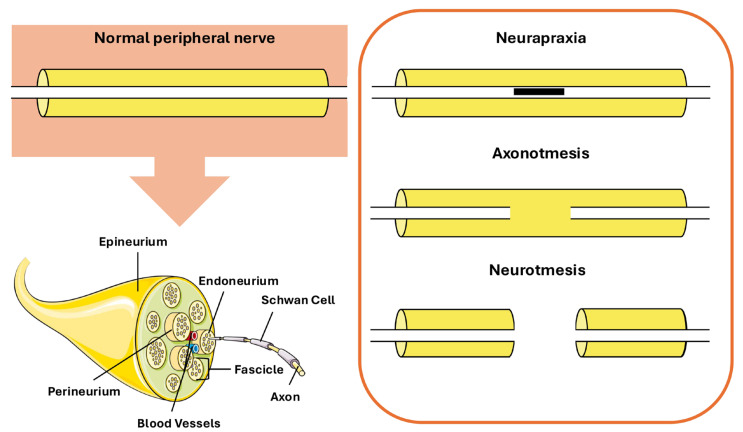
Different types of peripheral nerve lesions. Elements of this figure were adapted from Servier Medical Art, CC BY 4.0.

The classification of these injuries has evolved over time since Seddon’s 1942 framework that classifies nerve damage into three types: neurapraxia, axonotmesis, and neurotmesis [6,7] (Figure 1).

Neurapraxia involves transient functional loss with preservation of axonal integrity. Axonotmesis is represented by destroyed axons but with viable supportive connective tissues; recovery is slower because axons must regenerate. Neurotmesis is the most severe type where axons and their supportive connective tissues are completely severed and surgical intervention is usually necessary for recovery [8].

From a structural perspective, peripheral nerves exhibit a highly organized hierarchical microanatomy that underpins both their functional integrity and their appearance on imaging modalities [9]. Individual axons, either myelinated or unmyelinated, are grouped into fascicles and surrounded by the endoneurial compartment, which provides metabolic support and electrical insulation. Fascicles are enclosed by the perineurium, a multilamellar connective tissue sheath that forms a diffusion barrier and contributes to the blood–nerve barrier. Multiple fascicles are embedded within the interfascicular epineurium, a collagen-rich connective tissue matrix that accommodates blood vessels and provides mechanical protection, while the entire nerve is enveloped by the epineurium [10,11]. This histological organization has direct implications for nerve imaging, particularly ultrasound-based assessment. On sonography, peripheral nerves typically display a characteristic “honeycomb” appearance in transverse section, reflecting hypoechoic fascicles separated by hyperechoic interfascicular epineurium [12,13]. Recent ex vivo studies of the sciatic nerve have demonstrated that the relative amount and distribution of interfascicular epineurium critically influence fascicular differentiation and echogenic contrast on ultrasound, with nerves containing a higher proportion of interfascicular connective tissue exhibiting more clearly delineated fascicles [14]. These histological–sonographic correlations are highly relevant for translational research, as they provide a structural basis for interpreting in vivo and ex vivo imaging findings in experimental models of peripheral nerve injury and regeneration, according to each specific stage of damage (Table 1).

The aim of this review is to summarize selected recent literature on sciatic nerve regeneration, with a particular focus on functional assessment methodologies used in experimental animal models and how these methodologies are optimally deployed across the distinct phases of peripheral nerve degeneration and repair. Specifically, we draw on studies published within the last ten years to provide a critical evaluation of the strengths, limitations, and potential biases of the most employed methods for assessing locomotor recovery following peripheral nerve injury. In addition, this review highlights emerging trends and future therapeutic directions, emphasizing the need for standardized, phase-specific assessment tools that reflect both the biological progression of nerve repair and the functional demands of translational research. Finally, we address the persistent translational gap between animal models and clinical applications, underscoring the importance of aligning preclinical endpoints with clinically meaningful outcomes.

This review is primarily intended for basic and translational researchers working in peripheral nerve biology, neuroregeneration, and biomaterials, while also remaining accessible to clinician-scientists with an interest in experimental nerve repair. The detailed discussion of molecular and cellular mechanisms is included to provide a biological framework for interpreting phase-specific functional outcomes, whereas the emphasis on behavioral, electrophysiological, and translationally relevant assessment strategies is designed to facilitate the alignment of preclinical findings with clinically meaningful endpoints. The framework summarized in this review also served as an essential methodological foundation for our own preclinical investigation evaluating the regenerative potential of alpha-lipoic acid (ALA) (Thiossen) and calf blood hemodialysate (Actovegin), ensuring that our study employs phase-appropriate, biologically aligned, and translationally relevant outcome measures.

## 2. Methods

A structured literature search was conducted in PubMed for studies published between January 2015 and August 2025 using combinations of the following keywords: “sciatic nerve regeneration,” “sciatic functional index,” “Beam Walk,” “Rotarod,” “gait analysis,” “peripheral nerve injury,” “Wallerian degeneration,” “nerve conduits,” “electrical stimulation,” “exosomes,” “tacrolimus,” “inosine,” “platelet-rich plasma.” Additional searches targeted the molecular pathways relevant to degeneration and repair (e.g., SARM1–NMNAT2—sterile alpha and armadillo motif 1–nicotinamide mononucleotide adenyltransferase 2 axis, also named the Wallerian pathway, Schwann cell reprogramming, macrophage polarization). Reference lists from key reviews and primary studies were manually screened to identify additional articles. Eligible studies included rodent sciatic nerve injury models (crush, transection, gap, ligation, chronic constriction, chemical or thermal injury) that reported functional, sensory, electrophysiological, or histomorphometric outcomes. Exclusion criteria were non-sciatic models, studies without in vivo functional testing, systemic neuropathy models without traumatic lesions, and non–peer-reviewed sources. Clinical translation was assessed through a complementary search of ClinicalTrials.gov, restricted to the same time window.

In parallel, a computational analysis of ALA was performed to illustrate how molecular descriptors and docking data may complement functional and structural assessments in peripheral nerve research. Frontier molecular orbital energies, physicochemical descriptors, docking interactions with IL-6, TGF-β, and a growth factor receptor were obtained using HyperChem Professional (Release 8) and HEX softwares (version 8.0). Only ALA was evaluated computationally because it was the sole compound in our broader research project for which high-quality structural data, preliminary bioactivity rationale, and docking feasibility were available; other compounds lacked sufficient structural or mechanistic justification for inclusion. Together, these methodologies provided a foundation for synthesizing phase-specific functional assessment strategies and interpreting the translational relevance of emerging therapies in sciatic nerve regeneration.

## 3. Preclinical Models and Functional Assessment of Sciatic Nerve Injury

### 3.1. Preclinical Models of Sciatic Nerve Injury

Experimental peripheral nerve injury models have played a significant role in understanding the mechanisms of nerve damage, the regeneration process, and the efficacy of therapeutic interventions in neuroscience research [29]. These models are designed to reproduce clinically relevant types of nerve injury such as neurapraxia, axonotmesis, and neurotmesis. Rodents, especially rats and mice, are generally used as models since their nervous system is well characterized, they are cost-effective, and easy to handle [29,30]. Among existing models, the sciatic nerve is frequently chosen because of its size, accessibility, and critical role in both motor and sensory functions [31]. Its clear anatomical course makes it an ideal candidate for studies on nerve injury and regeneration. Generally, sciatic nerve injuries are induced by mechanical, chemical, or thermal damage [32,33,34,35,36,37,38] (Table 2). Crush injuries, which involve compression of the nerve without complete transection, model axonotmesis and simulate traumatic nerve injuries that occur humans [39]. Transection injuries involve complete severing of the nerve and model neurotmesis, allowing researchers to study the effects of complete discontinuity and the challenges associated with repair and regeneration [33,40]. Nerve ligation, in which the nerve is tightly tied off to induce ischemia, allows further insights into degeneration and subsequent regeneration [41]. The clinical relevance of such models lies in their ability to recapitulate key biological and molecular processes of nerve injury and repair [42]. By investigating these processes in animal models, it is possible to formulate and evaluate new therapeutic strategies, including nerve grafts, neurotrophic factors, biomaterials, and pharmacological treatments, before translation into clinical trials.

### 3.2. Functional Assessment in Preclinical Models of Peripheral Nerve Injury

A precise understanding of the mechanisms and classification of peripheral nerve injury is essential for developing appropriate therapeutic strategies and for accurately interpreting functional outcomes within preclinical research frameworks. Because degeneration and regeneration unfold through highly orchestrated temporal phases, functional assessment in animal models must be closely aligned with these biological events. Notably, failure to account for phase-specific changes in axonal physiology, Schwann cell behavior, or inflammatory dynamics can lead to misinterpretation of functional improvements or apparent treatment-related differences [52,53,54].

Within this context, behavioral and electrophysiological tools constitute indispensable instruments for preclinical assessment [55,56]. Among these approaches, the Sciatic Functional Index (SFI) remains the most widely applied quantitative measure of gait performance, providing a robust estimate of sciatic nerve integrity through footprint geometry and stance parameters [57]. However, because gait alone does not capture all dimensions of motor function, complementary behavioral assessments have gained increasing prominence. The Beam Walk test, for example, offers high sensitivity to deficits in balance, coordination, and fine motor control functional domains that often remain impaired even when SFI scores show substantial improvement [58]. This sensitivity makes the test especially valuable for detecting subtle deficits during the transition from partial to more refined motor recovery [58,59].

In parallel, the Rotarod test provides an automated and high-throughput assessment of motor coordination, fatigue resistance, and neuromuscular endurance [60]. Its utility is particularly pronounced during the late regenerative phase, when animals regain sufficient stability to perform continuous locomotion and when inter-group differences in coordination become more discriminating [60,61].

Importantly, sensory dysfunction is a major component of peripheral nerve injury in both clinical and preclinical settings [62]. Therefore, nociceptive assays such as the Hot Plate and Von Frey tests are an integral part of a comprehensive evaluation, as they quantify alterations in thermal and mechanical sensitivity that may arise from axonal degeneration, sensitization of nociceptive pathways, or aberrant regeneration [63,64,65]. These sensory endpoints provide essential complementary information that cannot be derived from motor tasks alone and are particularly relevant when evaluating therapies aimed at mitigating neuropathic pain or enhancing sensory reinnervation.

Beyond behavioral measurements, electrophysiological techniques serve as highly sensitive indicators of peripheral nerve integrity and recovery. Nerve conduction studies, compound muscle action potentials (CMAPs), and electromyography (EMG) allow for direct quantification of axonal continuity, conduction velocity, reinnervation efficacy, and neuromuscular junction function [66,67]. These metrics are critical for distinguishing between structural regeneration and compensatory motor strategies that may mask underlying deficits. When coupled with histological and ultrastructural analyses, including assessments of myelin thickness, axon density, macrophage activity, and Schwann cell morphology electrophysiological measurements bridge the gap between structural and functional outcomes [68,69,70].

In addition to behavioral, electrophysiological, and histomorphometric assessments, in vivo imaging modalities are increasingly recognized as essential tools for evaluating peripheral nerve integrity in a clinically relevant and non-invasive manner. High-resolution ultrasound, enables real-time visualization of peripheral nerves and has been shown to reliably depict intraneural architecture, including fascicular organization and interfascicular epineurium, with strong histological–sonographic correlation in both experimental and clinical settings [71,72,73,74]. This imagistic method allows longitudinal monitoring of nerve continuity, swelling, fibrosis, and neuroma formation. Complementary imaging methods, such as optical projection tomography and magnetic resonance microscopy, provide high-resolution three-dimensional characterization of nerve microstructure ex vivo and serve as valuable reference standards for validating in vivo findings [74]. Furthermore, advanced MRI techniques, including diffusion tensor imaging, further extend multiscale assessment by capturing axonal orientation, integrity, and myelination in vivo [70,75,76].

Taken together, the integration of behavioral, sensory, electrophysiological, and morphological assessments forms the cornerstone of modern preclinical peripheral nerve research. This multifaceted approach enables investigators to capture the full complexity of nerve degeneration and repair, identify treatment effects that may be phase-specific, and evaluate emerging therapeutic strategies with greater precision and translational value.

## 4. Pathophysiological Phases of Sciatic Nerve Injury

To rationally select functional tests and therapeutic interventions, it is essential to consider the temporal evolution of peripheral nerve injury, from acute axonal disintegration to late remyelination and possible maladaptive outcomes. In the following subsections, we summarize the main biological events of each phase and later correlate them to phase-appropriate assessment methods and treatments.

### 4.1. Acute Phase

The acute phase of peripheral nerve injury, unfolding within minutes to hours after the traumatic insult, is characterized by rapid axonal responses that precede the onset of classical Wallerian degeneration. It is dominated by abrupt disruptions in ionic gradients, cytoskeletal integrity, and mitochondrial homeostasis. A defining early event is a transient rise in intracellular calcium concentrations, observed in both spinal cord and optic nerve models [15,16,19]. This calcium influx initiates downstream molecular cascades, most notably the activation of the calcium-dependent cysteine proteases calpains. Once activated, calpains cleave structural and signaling proteins, leading to destabilization of the cytoskeleton and mitochondrial integrity and accelerating axonal fragmentation and the formation of characteristic degeneration bulbs [17]. Calpain-mediated proteolysis of collapsin response mediator protein-2 (CRMP-2), for example, has been identified as a key driver of acute axonal degeneration [18]. In addition to structural breakdown, calpains modulate early inflammatory signaling; in the dorsal root ganglion, they promote cytokine expression, thereby linking the immediate ionic dysregulation of injury to initiation of a pro-inflammatory microenvironment [77].

The SARM1–NMNAT2 axis has been defined as a crucial signaling pathway governing the cellular response to nerve injury [78]. Axonal degeneration appears to be orchestrated by SARM1, a neuronal NAD^+^ hydrolase that remains auto-inhibited under physiological conditions but becomes activated when the NMN/NAD^+^ ratio increases after injury [79]. Loss of the labile axonal enzyme NMNAT2, normally replenished from the soma, drives this imbalance; NMNAT2 synthesizes NAD^+^ and serves as a neuroprotective factor. Its depletion triggers SARM1-mediated NAD^+^ depletion, ATP collapse, calcium dysregulation, and subsequent axon fragmentation. The axon-protective phenotype of Wlds mice arises from ectopic stabilization of NMNAT1 in axons, sustaining NAD^+^ synthesis and preventing SARM1 activation [80].

Moreover, mitochondria are emerging as central mediators of acute axonal degeneration [81]. Disruption of mitochondrial membrane potential and opening of the mitochondrial permeability transition pore (mPTP) drive early metabolic collapse and structural disintegration. Pharmacological or genetic inhibition of cyclophilin D, a key mPTP component, protects axons against both mechanical transection and toxic insults, underscoring its role as a convergent effector of diverse injury pathways [82,83]. Mitochondrial impairment has been hypothesized to replicate all major steps of Wallerian degeneration by inducing NMNAT2 depletion and engaging the SARM1 cascade, thereby placing mitochondrial dysfunction upstream of programmed axon destruction and highlighting its pathogenic potential in peripheral nerve injury [84].

### 4.2. Subacute Phase

The subacute phase of peripheral nerve injury spans the period from several hours to days after the initial insult and corresponds to the onset and progression of Wallerian degeneration, meticulously described in Rotshenker’s schematic representation of the transition from intact to degenerating peripheral nerve [20]. At the molecular level, this phase is dominated by Schwann cell reprogramming, macrophage recruitment, and initiation of phagocytic clearance mechanisms.

During the early phase of Wallerian degeneration, the dynamic crosstalk between Schwann cells and macrophages constitutes the principal mechanism driving nerve repair, as Schwann cell-derived cytokines and chemokines orchestrate macrophage recruitment and polarization, while macrophages facilitate myelin debris clearance and promote Schwann cell proliferation and differentiation to further support axonal regeneration [85]. Upon peripheral nerve injury, Schwann cells undergo de-differentiation, transitioning from a myelinating phenotype to a more proliferative and motile state. De-differentiated Schwann cells lose their myelin-producing capabilities and instead begin to express neural repair-related molecules critical for regeneration [21,22]. Activation of c-Jun regulates expression of several trophic factors that promote nerve regeneration and Schwann cell de-differentiation [86]. During this de-differentiation process, Schwann cells also begin to express markers associated with phagocytosis, allowing them to participate actively in clearance of myelin debris even before significant macrophage infiltration occurs [26]. Concurrently, de-differentiated Schwann cells may upregulate heat shock proteins such as heme oxygenase-1 (HO-1), which are protective against oxidative stress and aid in cellular repair mechanisms [87]. Following nerve injury, Schwann cells produce cytokines such as interleukin-6 (IL-6) and macrophage chemoattractant protein-1 (MCP-1), functioning as chemoattractants that draw immune cells, especially macrophages to the site of injury [88]. Local production of serum amyloid A (SAA) by Schwann cells further illustrates their role in the inflammatory milieu during Wallerian degeneration, as SAA expression is implicated in induction of macrophage attractants, suggesting a feedback loop whereby Schwann cells actively recruit and regulate macrophage activity [89]. Resident macrophages outside the myelin sheath are activated, initiating local debris clearance and releasing chemokines to recruit circulating macrophages, which respond swiftly by migrating to the injured site [90]. Hematogenous macrophages subsequently adopt a pro-inflammatory M1 phenotype, amplifying the inflammatory cascade and promoting debris clearance through phagocytosis of myelin and axonal remnants for the first 3–4 days post-injury, thus concluding the subacute phase [91].

### 4.3. Early Regenerative Phase

At approximately 7–10 days after injury, the early regenerative phase of peripheral nerve repair begins, marking a critical transition from inflammation to regeneration. During this stage, progenitor-like Schwann cells adopt a reparative phenotype and organize into longitudinal multicellular columns known as Büngner bands, which provide both structural and biochemical guidance for regenerating axons [23]. These cells secrete key extracellular matrix components, including laminin-2 and laminin-8, and a wide array of neurotrophic and growth factors—such as glial cell line-derived neurotrophic factor (GDNF), brain-derived neurotrophic factor (BDNF), neurotrophin 3 (NT3), nerve growth factor (NGF), vascular endothelial growth factor (VEGF), and pleiotrophin—that collectively promote neuronal survival, axonal elongation, and remyelination [92]. In parallel, Schwann cells within the distal stump produce pro-inflammatory cytokines such as tumor necrosis factor (TNF)-α, IL-1α, IL-1β, leukemia inhibitory factor (LIF), and MCP-1, which serve to recruit circulating macrophages into the lesion site [24]. Initially, infiltrating macrophages exhibit a pro-inflammatory M1 phenotype responsible for clearing debris and secreting mediators that amplify the early inflammatory response. However, by approximately three days post-injury, a phenotypic shift occurs toward an anti-inflammatory M2 state that promotes resolution and tissue repair [93]. Alternative activation of macrophages into the M2 phenotype is mediated by IL-4, IL-13, transforming growth factor-β (TGF-β), immune complexes, and adenosine A_2_A receptor agonists [94]. Although Schwann cells themselves do not secrete classical M2-polarizing cytokines such as IL-4, IL-10, or IL-13, they play a crucial role in inducing M2 polarization, likely through direct cell–cell interactions and paracrine signaling [95].

In vitro studies demonstrate that Schwann cells and macrophages co-localize and maintain contact, leading to macrophage elongation and upregulation of CD163, a hallmark of M2 differentiation, though the precise mechanism remains under investigation [96]. As M2 macrophages increasingly populate the injury site, they release anti-inflammatory cytokines such as IL-10 and TGF-β, which modulate and balance the earlier pro-inflammatory M1 response, thereby restoring homeostasis and facilitating tissue regeneration [5]. These M2 macrophages express markers such as arginase-1 (Arg1), chitinase-like 3/Ym1, and mannose receptor C-type 1 (CD206/Mrc1), and their functional heterogeneity enables them to adapt to the evolving microenvironment [97]. Four subtypes of M2 macrophages have been identified, each contributing uniquely to regeneration. M2a macrophages mediate debris clearance and secrete IL-10 and TGF-β, promoting cell proliferation and migration. M2b macrophages enhance extracellular matrix (ECM) synthesis and angiogenesis, releasing IL-10 and VEGF-A. In turn, the presence of IL-10 and TGF-β promotes differentiation into M2c macrophages, responsible for tissue and ECM remodeling via upregulation of MMP-7, MMP-8, and TIMP-1 and release of arginase and Ym1 [98]. Finally, M2d macrophages support angiogenesis and restoration of blood flow, producing high levels of VEGF-A and promoting polarized vascular growth within the regenerating nerve [99]. This coordinated crosstalk between Schwann cells and macrophages defines the regenerative microenvironment characteristic of Wallerian degeneration. As macrophages clear myelin debris and secrete pro-angiogenic factors, Schwann cells migrate along newly formed capillaries, aligning into Büngner bands that serve as conduits for regenerating axons [100]. VEGF-A–mediated angiogenesis plays an important role in this process, improving oxygenation and supporting the metabolic demands of regeneration [101], while ECM proteins such as collagen I, collagen IV, and laminin facilitate Schwann cell adhesion and migration through integrin signaling [102]. Ultimately, the convergence of Schwann cell-driven structural organization and macrophage-mediated immune modulation ensures successful axonal regeneration and reinnervation of target tissues.

### 4.4. Late Regenerative Phase

In the final phase of peripheral nerve repair, typically extending over approximately 20 days and beyond depending on model severity, the regenerative environment undergoes a gradual transition from active repair to structural and functional stabilization [25]. As regeneration progresses, the inflammatory milieu gradually resolves, characterized by a decline in macrophage density following completion of debris clearance and ECM remodeling. A subset of macrophages persists as resident cells, secreting anti-inflammatory mediators, neurotrophic factors, and matrix-modulating enzymes to maintain a permissive microenvironment for axonal maturation, while excess macrophages undergo apoptosis or recirculate to lymphoid tissues [26]. Simultaneously, the blood–nerve barrier (BNB) is gradually reconstituted, with endothelial cells re-establishing tight junctions and restoring selective permeability [27]. Persistent endothelial dysfunction or incomplete BNB repair, often driven by sustained alterations in signaling pathways such as Hedgehog or thrombin-PAR1, can perpetuate vascular leakage and inflammatory sensitization, contributing to maladaptive outcomes including neuropathic pain and structural disorganization at the nodes of Ranvier [103,104].

At this stage, Schwann cells redifferentiate from their repair phenotype into a mature, myelinating state, synchronizing with axonal regrowth and target reinnervation [105]. They downregulate regeneration-associated genes such as c-Jun and upregulate myelin-associated proteins including myelin basic protein (MBP), protein zero (P0), and peripheral myelin protein 22 (PMP22) [106]. A pivotal aspect of this differentiation is production of laminin-2, a basement membrane glycoprotein essential for anchoring Schwann cells to axons and initiating concentric membrane wrapping [107]. Reformation of the myelin sheath restores saltatory conduction, enhancing speed and fidelity of electrical transmission and re-establishing functional connectivity between the peripheral and central nervous systems. However, incomplete or aberrant regeneration during this late phase may lead to maladaptive outcomes, compromising functional recovery. Failure of accurate axonal guidance or misalignment of regenerating fibers can result in neuroma formation, where disorganized axonal sprouts and proliferating Schwann cells form painful nodular structures [108]. Defective remyelination or persistent inflammation can cause chronic neuropathic pain, characterized by ectopic discharges and abnormal sensory signaling within the repaired nerve [109]. In some cases, inadequate reinnervation of target tissues leads to sustained motor or sensory deficits, even in the presence of structural continuity [110]. These maladaptive processes highlight the delicate balance between regeneration and repair resolution: while timely immune suppression and Schwann cell redifferentiation are essential for recovery, premature or incomplete transitions can lock the system in a dysfunctional state.

## 5. Phase-Specific Functional Assessment and Therapeutic Strategies

Understanding the temporal evolution of peripheral nerve injury is essential for selecting appropriate functional assessments and interpreting therapeutic effects in a biologically meaningful way. In this section, the four major phases of degeneration and regeneration, acute, subacute, early regenerative, and late regenerative are aligned with the functional tests most informative at each stage, together with therapeutic strategies whose mechanisms of action depend on the timing of intervention (Table 3).

### 5.1. Acute Phase: Electrophysiology, Sensory Testing, and Neuroprotection

The acute phase (minutes to hours after injury) is dominated by rapid ionic disturbances, calcium influx, calpain activation, mitochondrial depolarization, and activation of the SARM1–NMNAT2 pathway. At this early stage, gross locomotor behavior is typically insensitive to axonal compromise; classical motor tests such as the SFI, Beam Walk, or Rotarod do not reliably capture early deficits. Electrophysiological techniques, nerve conduction velocity, compound muscle action potentials, and F-waves are therefore the most informative, as they detect conduction block before gait abnormalities emerge [67,111]. Sensory assessments such as the Hot Plate or Von Frey tests may identify early hypo- or hyperalgesia, especially when small-fiber involvement is prominent [120,121,122].

Therapeutically, the acute phase represents a narrow but critical window for neuroprotection. Intraoperative electrical stimulation (20 Hz for 1 h) has been shown to enhance regeneration by increasing cyclic adenosine monophosphate (cAMP) and activating BDNF–TrkB, phospholipase C gamma (PLC-γ), phosphoinositide 3-kinase/protein kinase B (PI3K/AKT), and mitogen-activated protein kinase/extracellular signal-regulated kinase (MAPK/ERK pathways), thereby facilitating the shift from acute injury signaling to regenerative gene expression [123,124,125]. Early pharmacological interventions such as inosine, which activates cAMP–Mammalian sterile 20-like kinase-3b (Mst3b) signaling, or tacrolimus, which enhances neuron-intrinsic growth programs may likewise exert maximal benefit when administered shortly after injury [126,127,128,129]. These interventions aim to stabilize the injured axon, limit early degeneration, and prime the environment for subsequent regeneration.

### 5.2. Subacute Phase: Wallerian Degeneration, Sensory Dysfunction, and Immune Modulation

During the subacute phase, distal axons undergo Wallerian degeneration, Schwann cells dedifferentiate, and macrophages are recruited to initiate debris clearance. Electrophysiology remains central at this stage, as conduction failure and demyelination progress. Sensory testing becomes increasingly informative, capturing the onset of neuropathic pain or sensory loss [112,113,114]. Early motor tasks can be performed but require cautious interpretation: SFI measurements are often unstable due to irregular stepping patterns, while Rotarod performance may be confounded by anxiety or the animal’s reluctance to walk. Narrow-beam tasks and qualitative gait video analysis may reveal early placement errors, but they should be supported by electrophysiological and sensory outcomes.

Therapeutic strategies in this phase frequently target the immune microenvironment. IL-10-based modulation, as demonstrated by Golshadi et al., promotes macrophage polarization toward an M2 phenotype, improves Schwann-cell support, and restores conditions permissive for regeneration [130]. Platelet-rich plasma (PRP), rich in growth factors such as Platelet-Derived Growth Factor (PDGF), TGF-β, VEGF, NGF, and BDNF, has repeatedly demonstrated benefit in rat sciatic nerve injury when delivered through conduits or as an adjunct to surgical repair [131]. Early application of biomaterials, including decellularized nerve hydrogels or other natural and synthetic scaffolds, further stabilizes the lesion site and primes the extracellular matrix for later regenerative events.

### 5.3. Early Regenerative Phase: Return of Motor Function, SFI, Beam Walk, and Regenerative Scaffolds

By approximately 7–10 days post-injury, regenerating axons extend along Schwann-cell-derived Büngner bands, macrophages shift toward anti-inflammatory phenotypes, and angiogenesis supports metabolic demands. This is the first stage in which motor behavioral tests become truly informative [115]. The SFI is the most widely applied method, allowing quantitative longitudinal assessment of gait recovery [116]. Numerous studies have demonstrated its sensitivity to treatment effects, including those evaluating carbon nanotube conduits [132], polyethylene glycol (PEG)-fusion [133], botulinum toxin A [134], erythropoietin [135], and rosuvastatin [136]. Nonetheless, the SFI is susceptible to technical artifacts such as smeared footprints, joint contractures, or dorsal walking, and therefore benefits from complementary assessments.

The Beam Walk test provides a valuable counterpart by detecting subtle balance and coordination deficits that may not appear in gait metrics [58,117]. Electrophysiological improvements and histological indicators of remyelination and axonal density offer essential confirmatory evidence that behavioral recovery reflects genuine structural repair [137,138]. Therapeutic approaches that become effective during this stage include bioengineered scaffolds, growth-factor-loaded conduits, conductive and piezoelectric nerve guides, localized tacrolimus delivery, exosome-loaded hydrogels, and small molecules such as inosine. These strategies promote axonal elongation, Schwann-cell differentiation, angiogenesis, and remyelination, and their functional benefits are best detected during this early regenerative window [139,140,141].

### 5.4. Late Regenerative Phase: Consolidation, Functional Refinement, and Chronic Outcomes

Weeks to months after injury, axonal maturation and remyelination continue, neuromuscular junctions are re-established, and the blood–nerve barrier is restored. However, persistent deficits, neuroma formation, or chronic neuropathic pain may emerge, underscoring the need for multimodal assessment [110,142]. The SFI may approach baseline even when deficits remain, as compensatory strategies can mask impairments. The Beam Walk test retains high sensitivity to subtle coordination and postural abnormalities and is therefore valuable for detecting incomplete recovery. The Rotarod test becomes particularly informative in this stage, revealing differences in motor coordination, balance, and fatigue resistance once animals regain stable gait [116,118]. Electrophysiology, histomorphometry, and measures of muscle reinnervation (including target muscle mass and neuromuscular junction morphology) provide essential structural correlates [119,143,144,145,146].

Therapeutically, late-phase interventions aim to consolidate regeneration and prevent maladaptive outcomes. Bioengineered conduits continue to influence Schwann-cell differentiation and extracellular-matrix remodeling, while immunomodulation strategies help maintain a favorable repair environment [147,148,149]. Small molecules such as cnicin, which modulates the tubulin tyrosination/detyrosination cycle, have demonstrated late-phase improvements in motor outcomes and electrophysiological recovery [150,151]. Rehabilitation paradigms, such as treadmill training, further synergize with molecular therapies.

Collectively, late-phase assessment requires integration of behavioral, electrophysiological, sensory, and structural endpoints to distinguish true recovery from compensation and to evaluate the durability and completeness of therapeutic effects. This multimodal approach aligns closely with clinically relevant outcomes, supporting the translation of promising interventions into human studies.

## 6. Computational Analysis and Molecular Docking of ALA

Although this review is primarily focused on phase-specific functional assessment and therapeutic strategies in sciatic nerve injury, the mechanistic understanding of candidate neuroprotective and pro-regenerative agents increasingly relies on complementary in silico approaches. In this context, we included a brief computational characterization of ALA to illustrate how electronic descriptors and molecular docking can provide additional insight into potential interactions with key inflammatory and regenerative mediators, such as IL-6 and TGF-β. This targeted analysis links molecular properties of a candidate compound to signaling pathways that modulate inflammation, repair, and tissue remodeling. α-Lipoic acid was selected for in silico analysis because it represents a chemically well-defined small molecule and constitutes the principal active ingredient of Thiossen, a formulation used in experimental and clinical contexts for its antioxidant and neuroprotective properties.

### 6.1. Electronic Structure and Molecular Descriptors of ALA

**Figure 2:**
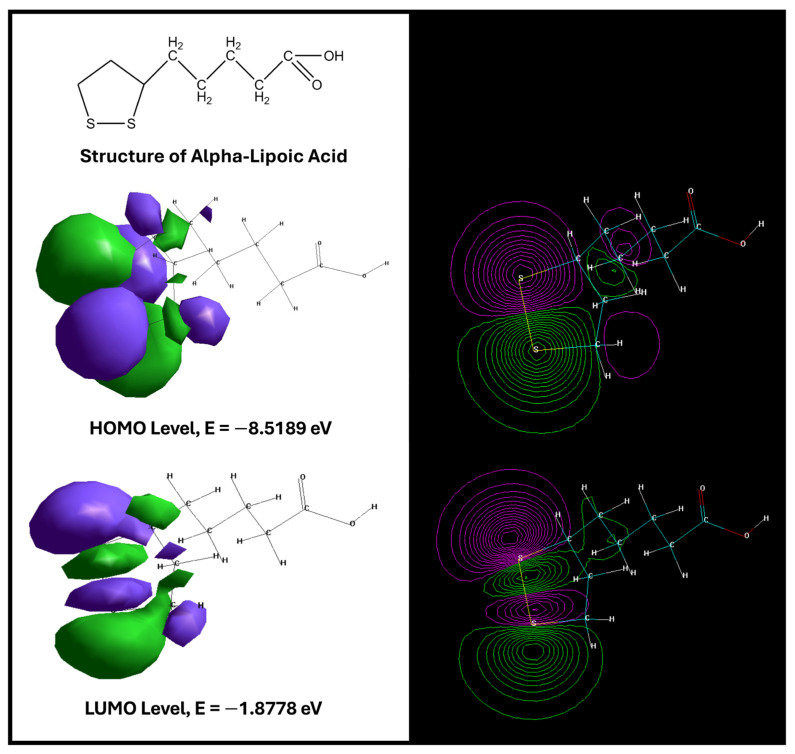
Structure of ALA. HOMO and LUMO energy levels of the analyzed compound [152].

The optimized structure of ALA is shown in Figure 2. Frontier molecular orbital analysis revealed a HOMO energy of –8.5189 eV and a LUMO energy of –1.8778 eV (Figure 2).

The relatively low HOMO energy indicates a reduced tendency to donate electrons, suggesting good stability of the electron-rich regions, while the LUMO energy is consistent with a moderate capacity to accept electrons. The HOMO–LUMO energy gap (ΔE = 6.6411 eV) is comparatively large, a feature typically associated with chemically stable molecules exhibiting lower intrinsic reactivity and higher “hardness.” Such stability may favor selective rather than promiscuous interactions with biological targets, which is relevant when considering chronic administration in neuroprotective or regenerative settings.

Additional molecular descriptors further refine the pharmacochemical profile of ALA (Table 4). The compound displays a relatively large surface area (377.56 Å^2^) and volume (616.4 Å^3^), features that may influence steric complementarity within protein binding pockets. Its hydration energy (–7.07 kcal/mol) reflects modest solvation stability, while a logP value of 1.78 suggests moderate lipophilicity, compatible with balanced membrane permeability without excessive hydrophobicity. The refractivity (54.29 Å^3^) and polarizability (21.4 Å^3^) indicate a capacity for electronic deformation that may enhance binding affinity in polarizable environments, such as cytokine or growth-factor receptors. Finally, a dipole moment of 4.256 D denotes moderate polarity, potentially favoring hydrogen bonding and electrostatic interactions at the ligand–protein interface.

Taken together, these descriptors provide a coherent picture of ALA as a chemically stable, moderately lipophilic, and electronically adaptable small molecule with a plausible profile for interaction with protein targets relevant to nerve injury and regeneration.

### 6.2. Docking of ALA to IL-6, Growth-Factor Receptors, and TGF-β

To explore potential mechanistic links between ALA and pathways implicated in peripheral nerve injury, molecular docking studies were conducted using IL-6 (PDB: 2ARW), a representative growth factor receptor (4XPJ), and TGF-β (1B6C) as targets (Table 5). Docking scores were –173.02 kcal/mol for IL-6, –180.71 kcal/mol for the growth-factor receptor, and –191.89 kcal/mol for TGF-β, indicating favorable binding geometries across all three proteins, with the most stable predicted interaction for TGF-β.

**Figure 3:**
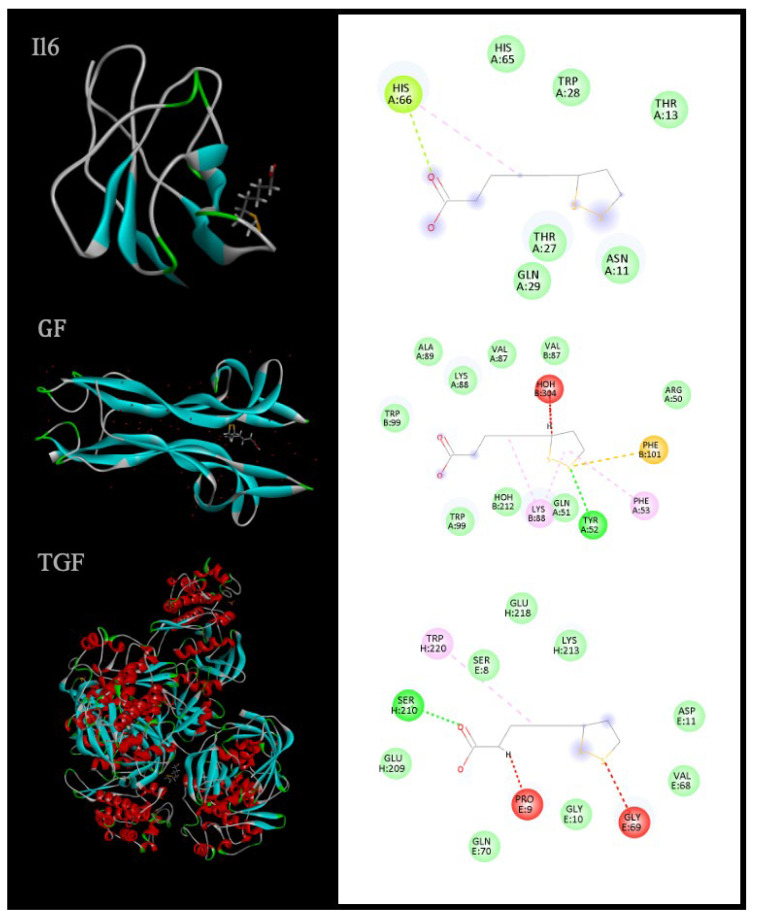
3D and 2D representations of ligand–receptor interactions highlight spatial orientation and key contact residues, providing insight into molecular binding mechanisms [153].

Visual inspection of 3D and 2D ligand–receptor interaction maps (Figure 3) revealed that ALA adopts well-defined binding poses in proximity to functionally relevant residues, stabilized by a combination of hydrogen bonds, hydrophobic contacts, and electrostatic interactions. While docking scores do not equate to direct biological efficacy, they support the hypothesis that ALA can engage with cytokine and growth-factor pathways that orchestrate inflammation, extracellular matrix remodeling, and tissue regeneration—processes central to Wallerian degeneration and subsequent repair.

Growth factors and cytokines such as IL-6 and TGF-β are key regulators of the balance between inflammation and regeneration. IL-6 is rapidly upregulated after tissue injury and can exert both pro-inflammatory and pro-regenerative effects depending on receptor context and signaling mode, whereas TGF-β predominantly mediates immunomodulation, matrix synthesis, and tissue remodeling.

The ability of ALA to adopt favorable binding conformations within these targets suggests a potential to modulate the inflammatory–regenerative axis at multiple levels. Although experimental validation is required, these in silico findings provide a mechanistic hypothesis that complements the functional and histological endpoints used in preclinical sciatic nerve models.

### 6.3. Rationale for Restricting Computational Analysis to ALA

A similar computational analysis was not extended to the other agents discussed in this review for both methodological and conceptual reasons. First, ALA is a well-defined small molecule with a precisely known structure, which makes it suitable for quantum-chemical calculations and receptor docking using standardized software and protocols. By contrast, preparations such as Actovegin are complex, multicomponent biological products with incompletely characterized or heterogeneous molecular compositions. For such agents, assigning a single representative structure for HOMO–LUMO analysis or docking would be methodologically unreliable and potentially misleading. For these reasons, in silico characterization was deliberately restricted to ALA, where the structural definition and parameterization allowed for a coherent, reproducible analysis that could be meaningfully integrated with the broader framework.

## 7. Comparative Analysis of Functional Tests and Translational Value

A wide range of behavioral, sensory, ultrasonographic, imaging and electrophysiological methods has been developed to evaluate functional recovery following peripheral nerve injury, each capturing different aspects of motor, sensory, and neuromuscular performance. When comparing these tests across all phases of degeneration and regeneration, it becomes evident that no single assessment method provides a complete picture of functional restoration. Instead, each tool possesses distinct strengths and limitations that make it more or less suitable depending on the biological stage of recovery and the specific functional domain under investigation (Table 6)., these observations underscore a central principle in peripheral nerve research: no single functional test can adequately capture the full temporal and multidimensional landscape of nerve regeneration. A phase-specific combination is therefore required—electrophysiology and sensory testing in the acute/subacute phase; SFI, Beam Walk, and electrophysiology during early regeneration; and SFI, Beam Walk, Rotarod, morphology, and pain behavior assessments during late regeneration.

## 8. Overview of Novel Treatment Approaches

Peripheral nerve injury triggers a complex cascade of degenerative and regenerative events. Surgical repair can yield suboptimal functional recovery due to the limited intrinsic regeneration capabilities and the specific post-injury microenvironment. Recent research has focused on therapies that modulate specific molecular pathways to enhance nerve regeneration. Intraoperative electrical stimulation (ES) is an adjunct therapy that can be applied in a variety of ways in terms of equipment type, frequency and duration [172,173,174], with the 1 h at 20 Hz protocol being the most widely adopted approach [173,175]. Applied to the proximal nerve segment, it induces membrane depolarization and Ca^2+^ influx, elevates cAMP, and up-regulates BDNF-trkB which activates phospholipase PLC-γ, PI3K/AKT, and MAPK/ERK pathways that promote cytoskeletal remodeling, growth cone stimulation, and regeneration-associated gene transcription for accelerated recovery [173,176]. However, the optimal ES parameters are still being determined, and current use of ES is not yet widespread standard of care.

Bioscaffolds are engineered biomaterials that provide structural support and biochemical guidance for peripheral nerve regeneration. By tailoring their composition and architecture, they establish a pro-regenerative microenvironment that directs axonal growth, enhances Schwann-cell activity, and modulates inflammation [173,177,178]. Natural biomaterials such as chitosan, silk, fibroin, keratin, decellularized extracellular matrix (dECM)-based natural polymers have not proven their superiority over autografts [173,179]. Synthetic materials like poly l-lactic acid (PLLA), poly l-lactic acid-co-ε-caprolactone (PLCL), and poly vinylidene fluoride (PVDF) and its copolymers with trifluoroethylene (PVDF-TrFE) offer mechanical stability. The inclusion of bioactive molecules such as IGF-1 stimulate neurite extension through MAPK and PI3K pathway activation [173,180] and extracellular matrix-derived (ECM-derived) components stabilize injury site, support regeneration and angiogenesis [173,181]. Also, a study by Liu et al. (2024) showed that a PLCL/SF/NGF@TA-PPy-RGD conduit (poly(L-lactic-co-ε-caprolactone)/silk fibroin/nerve growth factor@tannic acid-polypyrrole-L-Arg-Gly-L-Asp) improved axon remyelination, muscle recovery, and angiogenesis, achieving regeneration comparable to autologous nerve transplantation in a rat sciatic nerve defect model [182].

Advanced conductive and piezoelectric designs add localized electric cues, amplifying calcium influx and neurotrophic signaling to accelerate nerve repair. Unlike ES, which depends on external electrodes or implanted stimulators, piezoelectric mechano-electrical stimulation (MES) integrates the electrical source directly into the nerve conduit. A study by Tai et al. (2023) [183] investigated this approach in a mouse model of sciatic nerve transection with a 15 mm gap. A scaffold composed of P(VDF-TrFE) piezoelectric nanofibers generated localized electrical fields in response to mechanical impulses. This led to complete axonal reconnection, enhanced Schwann-cell differentiation, and robust remyelination [183]. A 2024 cell biomaterials study introduced a biohybrid neurodevelopment-inspired self-evolving neural scaffold (ND-SENS) combining piezoelectric PLLA fibers, hydrogel, and stem cells. Activated by ultrasound, it stimulates Ca^2+^-mediated PI3K/AKT and Ras/MAPK signaling, enhancing Schwann-like differentiation, neurotrophic secretion, and axonal regeneration. In a rat sciatic nerve injury model, ND-SENS achieved nerve repair comparable to autologous transplantation [184].

Another therapeutic option being explored is tacrolimus (FK506) which enhances neuron-intrinsic growth signaling through mechanisms distinct from its immune effects [185]. Because of its significant adverse effects in systemic administration, localized pharmacology is being studied. A 2024 preclinical study demonstrated a tacrolimus-eluting nerve guidance conduit that increased axonal regeneration across a 15 mm-size gap in sciatic nerves of rats [186]. PRP emerges as another biologic therapy for peripheral nerve lesions [187]. PRP is a concentrate of platelets obtained from the patient’s own blood, delivering a multitude of growth factors (PDGF, TGF-β, VEGF, NGF, BDNF, etc.), together with anti-inflammatory cytokines, promoting angiogenesis and regeneration [188]. In rat sciatic nerve injury models, both Yuan et al. and Zavala et al. demonstrated that incorporating PRP into repair approaches (within a collagen/chitosan conduit, as an adjunct to standard suture repair or combined with curcumin) significantly enhanced recovery compared to control groups [189,190]. Golshadi et al. (2023) [130] demonstrated that immune dysregulation is a major barrier to regeneration in delayed sciatic nerve repair. Their work highlights that exogenous IL-10, a potent anti-inflammatory cytokine, restores the regenerative microenvironment by polarizing macrophages, enhancing axonal growth and neuromuscular junction reformation [130].

Another potential new therapeutic option is engineered exosome-loaded decellularized-nerve hydrogels for PNI repair, presented by Liu et al. (2024) [191]. Exosomes were complexed with polyethylenimine (PEI) before integrating them into the DNH, creating polyplex hydrogels (dExo-loaded pDNH). In a rat sciatic nerve crush model, they significantly improved remyelination and motor/sensory function recovery compared to controls [191]. A particular target is the tubulin tyrosination/detyrosination cycle that regulates growth-cone dynamics. Vasohibin induces microtubule detyrosination, which slows recovery. Cnicin, a sesquiterpene lactone, inhibits the vasohibin axis, enhancing axonal regeneration and recovery after sciatic crush in rodents, in both oral and parenteral administration [150,151]. Inosine, a purine nucleoside, is another potential adjuvant for peripheral nerve injury. In a mouse sciatic nerve transection model repaired with a polylactic acid (PLA) conduit, inosine (1 h post-injury, daily × 7 days, intraperitoneal) significantly improved regeneration. Through the cAMP- Mst3b path, it increased myelinated fiber count, neurofilament high chain (NFH), MBP expression, and adenosine A2 (A2A) receptor activation, leading to enhanced axon remyelination, nerve conduction velocity, and motor-sensory recovery [192]. Another recent study showed that inosine and treadmill training (10 min, 3 times weekly beginning day 7 post-injury) synergistically accelerated functional recovery, improved electrophysiological outcomes, and enhancing myelination [193].

Despite extensive preclinical innovation, several therapeutic strategies which showed robust biological or histological efficacy have failed to translate into consistent clinical practice. For instance, neurotrophic factor-based approaches (e.g., NGF, BDNF, GDNF) reliably enhance axonal sprouting and myelination in rodent sciatic nerve models but have demonstrated limited functional benefit and unacceptable sensory side effects, including pain and hyperalgesia, in clinical settings [194,195]. Systemic tacrolimus enhances neuron-intrinsic growth signaling experimentally, yet its clinical use is precluded by dose-limiting toxicity, prompting localized delivery strategies that mainly remain preclinical [196,197]. Cell-based therapies, including Schwann cells, mesenchymal stem cells, and induced pluripotent stem cell-derived constructs, often improve histomorphometric and electrophysiological parameters but yield variable and frequently modest motor recovery, particularly in long-gap injuries, with additional challenges related to cell survival, heterogeneity, and importantly regulatory concerns [198,199,200]. Similarly, many synthetic nerve conduits and growth-factor-loaded scaffolds perform well in short-gap or crush models but fail to achieve functional equivalence to autologous nerve grafts in clinically relevant long-gap defects, reflecting scale and timing dependent limitations [201,202]. Furthermore, electrical stimulation, despite strong mechanistic rationale and reproducible acceleration of early axonal outgrowth, has not been widely adopted clinically due to protocol variability, logistical constraints, and inconsistent long-term functional gains [124,203]. Together, these examples suggest that biological regeneration does not necessarily equate to functional restoration and underscore the importance of multimodal functional assessment.

## 9. Analysis of Publication Trends and Current Research Gaps

The field of sciatic nerve injury has shown a steady trend in the number of publications over the past five years, with an annual volume ranging from 519 to 701 articles and a total of 2658 papers according to the PubMed database. This steady flow reflects growing interest in understanding peripheral nerve injury and in developing innovative therapeutic strategies. Despite this high publication rate, a closer analysis reveals an extremely low number of studies classified as “clinical trials” or “clinical studies”. This suggests a gap in translation of preclinical work into clinical practice, driven by variability in experimental designs, differences between preclinical models and real human conditions, and difficulties in accurately reproducing those conditions. The lack of standardized evaluation protocols and the complexity of peripheral nerve repair further hinder direct application of preclinical results.

A PubMed search using the keyword “sciatic nerve injury” over the past five years yields 2658 entries, including 16 meta-analyses, 108 reviews, 38 systematic reviews, and 19 manuscripts discussing clinical trials related to SNI. Examination of ClinicalTrials.gov identified only 20 clinical studies at various stages, of which one was “Not yet recruiting” and none were currently recruiting or actively enrolling by invitation. Thirteen studies were completed, three terminated, and three listed with “Unknown” status. No trials were categorized as active but not recruiting, suspended, or withdrawn; none were registered under expanded-access or treatment IND categories. The limited number of active or recruiting trials, coupled with the absence of recently reported results, highlights a stark translational gap between preclinical and clinical realms. This gap underscores the need for innovative methodologies and collaborative frameworks that link phase-specific biology, functional testing, and clinically meaningful outcomes to accelerate development of effective interventions for sciatic nerve injury.

A significant contributor to the translational gap in sciatic nerve research could lie in fundamental anatomical, temporal, and biological differences between preclinical models and human peripheral nerve repair. For instance, in rodents, regeneration distances are short. Moreover, target muscles remain relatively close to the injury site, and axonal regrowth rates (≈2–4 mm/day) often allow reinnervation before irreversible motor endplate degeneration occurs. On the other hand, in humans, regeneration must frequently span tens of centimeters at slower effective rates (≈1 mm/day), greatly increasing the likelihood of denervation-induced muscle atrophy and loss of functional targets [204]. Rodents also exhibit greater functional redundancy and plasticity within motor units, enabling compensatory strategies that can normalize gait-based metrics such as the Sciatic Functional Index despite incomplete or misdirected reinnervation, whereas human motor recovery requires precise, long-distance axonal targeting for meaningful functional restoration [205,206]. In addition, immune and inflammatory responses also differ substantially across species. Rodents display a more rapid and strictly regulated transition from pro-inflammatory to pro-regenerative macrophage phenotypes, while prolonged or dysregulated inflammation is common in human injuries, particularly in the context of aging metabolic disease [207,208,209]. Factors such as differences in nerve caliber, fascicular organization, blood–nerve barrier dynamics, and Schwann-cell longevity further influence remyelination efficiency and long-term conduction fidelity. Together, these factors mean that therapies capable of accelerating early regeneration or improving short-distance repair in rodents may fail to produce durable, functionally relevant recovery in humans.

## 10. Future Research and Discussion

Future research on sciatic nerve regeneration should prioritize the development and integration of advanced, multifunctional assessment tools that provide highly accurate, phase-specific data and reduce the discrepancy between preclinical results and clinical application. The translational gap is particularly evident in neurodegenerative and neurotrauma research, where purely molecular and cellular indicators are insufficient to predict real-world functional recovery. The complexity of symptoms emphasizes the need for more elaborate functional investigations. The increasing sophistication of therapeutic approaches—bioengineered scaffolds, gene delivery, exosomes, piezoelectric conduits, and immunotherapies—requires parallel advancement in outcome measures. State-of-the-art technologies, such as advanced imaging (in vivo MRI, diffusion tensor imaging, two-photon microscopy), artificial intelligence (AI), and machine learning (ML), could substantially increase the accuracy and efficiency of nerve regeneration assessments by extracting subtle features from gait, kinematics, and histology [210,211,212]. Expanding research to larger animal models that more closely mimic human injury patterns, age, comorbidities, and recovery trajectories is also crucial. While rodent models have been instrumental, their translational power is limited by differences in anatomy, regenerative capacity, and functional complexity. Incorporating aged animals, metabolic comorbidities, or models of polytrauma can further improve external validity.

New trends in therapeutic strategies, bioengineered scaffolds, growth factor delivery systems, nerve conduits, stem cell therapies, and neuro-electronic interfaces, are beginning to play key roles in nerve regeneration [213,214,215,216]. Biomaterials can reconnect nerves by creating supportive environments that guide regeneration and prevent scar formation [217,218]. Stem cells modulate immune responses and secrete neurotrophic factors, stimulating axonal regrowth and remyelination [216,219,220]. Neuro-electronic interfaces and bioelectronics enable real-time monitoring and targeted stimulation of nerves, potentially optimizing rehabilitation. Genomic and molecular profiling of nerve injuries holds promise for identifying biomarkers and stratifying patients for personalized therapies [221]. At the same time, immune modulation—controlling macrophage polarization, microglial activation, and cytokine signaling—offers avenues to promote regeneration while limiting chronic inflammation [222,223]. However, without standardized and sensitive behavioral analyses, even the most sophisticated therapies will face translational barriers. Current behavioral tests (SFI, Beam Walk, Rotarod) remain indispensable but must be embedded in a phase-specific, multimodal framework and, where possible, augmented by automated 3D kinematics, AI-assisted gait analysis, and clinically aligned outcomes [212,224]. Cohort-related parameters (age, sex, weight, comorbidities) should be chosen to mirror at-risk human populations, increasing the validity and translational value of preclinical findings.

## 11. Conclusions

In conclusion, sciatic nerve regeneration research has made significant progress, with numerous innovative therapeutic strategies emerging, including bioengineered constructs, stem cell therapies, viral vectors, neuro-electronic interfaces, exosome-based approaches, and targeted immunomodulation. These interventions offer considerable potential to improve outcomes for patients with peripheral nerve injuries. Despite these advances, a substantial gap in the development and application of modern, statistically robust, and phase-appropriate behavioral analysis tools to assess functional recovery in preclinical settings is still present. Current methodologies, although valuable, often fail to fully capture the temporal and multidimensional complexity of nerve degeneration and regeneration. Nerve regeneration depends not only on anatomical and biological repair but also on the restoration of function. It is therefore vital that future research prioritizes integration of sophisticated, highly replicable, and standardized assessment methods that are explicitly matched to the acute, subacute, early regenerative, and late regenerative phases. Multimodal batteries combining SFI, Beam Walk, Rotarod, electrophysiology, morphology, pain behavior, and advanced imaging, possibly enhanced by AI-based analysis, are likely to yield more reproducible and clinically relevant datasets. As research increasingly focuses on personalized medicine and advanced therapies, a parallel evolution of functional assessment tools is indispensable to ensure efficient translation into clinical applications.

## Figures and Tables

**Table 1:** Stages of Nerve Damage.

Stage of Nerve Damage	Time Window	Key Features	Ref.
1. Acute Phase	Minutes → Hours	Immediate loss of conduction, ionic disturbances, early axonal instability.	[15,16,17,18,19]
2. Subacute Phase (Wallerian Degeneration)	Hours → Days	Breakdown of distal axons, Schwann cell activation, initiation of inflammatory response.	[19,20,21,22]
3. Early Regenerative Phase	~7–14 Days	Axonal sprouting begins, Schwann cells guide regenerating fibers, inflammation shifts toward repair.	[3,23,24]
4. Late Regenerative Phase	Weeks → Months	Axonal maturation, remyelination, restoration of neuromuscular connections.	[3,25,26,27,28]

**Table 2:** Preclinical Animal Models of Peripheral Nerve Injury.

Injury Model	Mechanism/Method	Corresponding Clinical Condition	Pathophysiological Features Reproduced	Research Applications	Ref.
Crush Injury (Axonotmesis Model)	Controlled compression of the sciatic nerve using forceps, clamps, or standardized pressure devices	Traumatic nerve compression (e.g., limb trauma, entrapment injuries)	Axonal disruption with preserved connective tissue sheaths Wallerian degeneration distal to injury Schwann cell activation and remyelination	Study of axonal regeneration kinetics Evaluation of neuroprotective drugs Testing scaffolds and growth factor delivery Standard model for functional recovery studies	[32,34,39,43,44]
Transection Injury (Neurotmesis Model)	Complete severing of the nerve; often followed by immediate or delayed microsurgical repair	Severe lacerations, surgical injury, penetrating trauma	Total discontinuity of axons and connective tissue layers Requires axonal bridging and guided regeneration High risk of neuroma formation	Assessment of nerve repair techniques (sutures, grafts, conduits) Evaluation of biomaterials and nerve guidance scaffolds Long-gap repair studies	[32,34,44]
Nerve Ligation (Ischemic/Compressive Model)	Tight ligature around the nerve inducing ischemia, inflammation, and conduction block	Chronic nerve compression, ischemic neuropathies	Ischemia-induced axonal degeneration Persistent inflammation and neuropathic pain Sensory hypersensitivity and demyelination	Studies of neuropathic pain mechanisms Evaluation of anti-inflammatory or analgesic therapies Examination of ischemia-driven degeneration	[32,41,45]
Chemical Injury	Exposure to agents such as alcohol, phenol, or chemotoxins	Chemical neuropathies, iatrogenic nerve damage	Direct neurotoxicity and demyelination Schwann cell dysfunction Variable axonal loss depending on agent	Modeling toxic neuropathies Testing neuroprotective compounds and antioxidants	[29,37,46,47]
Thermal Injury	Controlled application of heat or cold to induce localized nerve damage	Burn injury, frostbite, thermal trauma	Protein denaturation, axonal necrosis Microvascular injury and inflammatory infiltration	Study of severe nerve degeneration mechanisms Evaluation of regenerative therapies under harsh injury conditions	[35,36,48]
Chronic Constriction Injury (CCI)	Multiple loose ligatures around the sciatic nerve	Chronic neuropathic pain conditions	Mixed axonal injury and inflammation Mechanical allodynia and hyperalgesia	Neuropathic pain research Screening analgesics and anti-inflammatory agents	[32,49,50]
Gap Injury Model	Surgical removal of a nerve segment creating a measurable defect	Long-gap nerve loss from trauma or tumor resection	Loss of nerve continuity requiring bridging structure Demands robust axonal elongation and guidance	Testing nerve grafts, conduits, and bioengineered scaffolds Investigation of factors influencing long-distance regeneration	[38,51]

**Table 3:** Phase-Specific Functional Assessment and Therapeutic Strategies in Peripheral Nerve Injury.

Phase (Timing)	Dominant Microenvironment	Most Informative Functional Readouts	Less Informative/Use with Caution	Phase-Targeted Therapeutic Strategies	Clinical Analogue/Translational Anchor	Ref.
Acute Phase (minutes–hours)	Ionic dysregulation (Na^+^/Ca^2+^ influx) Calpain activation, cytoskeletal breakdown Mitochondrial depolarization, mPTP opening SARM1–NMNAT2 pathway activation, early axonal fragmentation	Nerve Conduction Velocity (NCV), CMAP, F-waves (detect conduction block) EMG for acute denervation Thermal/mechanical nociceptive tests	SFI, Beam Walk, Rotarod (motor output often appears normal) Open-field scoring without electrophysiology	Intraoperative electrical stimulation (20 Hz, 1 h) Acute neuroprotection (inosine, tacrolimus) Anti-excitotoxic and mitochondrial-protective strategies	Intraoperative nerve monitoring Early EMG/NCV Acute sensory disturbances after trauma	[15,16,17,18,19,67,83,111]
Subacute Phase/Wallerian Degeneration (hours–days)	Schwann cell de-differentiation (c-Jun) Macrophage recruitment (M1) Debris clearance (myelin + axon) IL-6, TNF-α, MCP-1, SAA upregulation	Serial NCV/CMAP decline Von Frey, dynamic plantar, thermal tests Early gait and paw placement via video Narrow Beam Walk for early slips	SFI (unstable footprints, dorsal stepping) Rotarod (anxiety/refusal confounds) Single time-point behavior	IL-10 immunomodulation (M2 shift) Platelet-rich plasma (PRP) Early implantation of conduits, hydrogels	Subacute EMG/NCV tracking Early neuropathic pain symptoms Sensory loss/allodynia	[19,20,21,22,112,113,114]
Early Regenerative Phase (~7–14 days)	Büngner band formation M2 macrophage polarization (IL-10, TGF-β, VEGF-A) ECM remodeling (laminin, collagen) Onset of axonal elongation	SFI for longitudinal gait recovery Beam Walk (slips, step accuracy) NCV/CMAP improvement Optional 3D gait/kinematic analysis	Rotarod (still inconsistent) Single-endpoint SFI without correlates	Bioengineered nerve guides (PLCL, chitosan, dECM) Conductive/piezoelectric conduits (PVDF-TrFE, PLLA) Local tacrolimus delivery Exosome-loaded hydrogels Inosine + treadmill training	Early postoperative gait/balance recovery EMG evidence of early reinnervation	[3,23,24,115,116,117]
Late Regenerative Phase (weeks–months)	Schwann cell redifferentiation (MBP, P0, PMP22) Blood–nerve barrier repair NMJ maturation Risk of neuroma, chronic pain	SFI (gross recovery) Beam Walk (fine motor deficits) Rotarod (coordination, endurance) NCV/CMAP plateau Histomorphometry (fiber density, g-ratio) Muscle mass/force Chronic pain tests	Only SFI (may be near-normal despite deficits) Behavioral tests without structural correlates	Long-term conduit/scaffold outcomes Anti-inflammatory and anti-neuroma strategies Chronic-phase strengthening + rehab	Long-term gait and balance tests Neuropathic pain scales Late EMG/NCV, muscle imaging	[3,25,26,27,28,116,118,119]

**Table 4:** Molecular descriptors of ALA.

Compound	SA, A^2^	V, A^3^	E_h_, kcal/mol	logP	R_M_, A^3^	α, A^3^	μ, D
**1**	377.56	616.4	−7.07	1.78	54.29	21.4	4.256

(SA—surface area; V—volume; E_h_—hydration energy; R_M_—refractivity; α—polarizability; μ—dipole moment).

**Table 5:** Docking results of ALA with different receptors [https://hex.loria.fr, accessed on 10 December 2025].

Receptor	Energy (kcal/mol)
IL6 2ARW	−173.02
Growth factor 4XPJ	−180.71
Tgf beta 1B6C	−191.89

**Table 6:** Comparison of functional assessment tools.

Test	Primary Strengths	Weaknesses/Limitations	Additional Advantages/Translational Notes	Ref.
Sciatic Functional Index (SFI)	Gold standard for gait analysis Quantifies sciatic nerve–specific deficits (toe spread, print length) Useful for longitudinal monitoring	Low sensitivity in acute/subacute phases Footprint smearing, contractures, autotomy reduce reliability Compensation may mask deficits	Strong correlation with structural regeneration when combined with histology Valuable for evaluating crush or transection repair outcomes	[57,154,155,156]
Beam Walk Test	Detects subtle balance and coordination deficits Sensitive to small improvements often missed by SFI Assesses cerebellar–proprioceptive integration	Time-consuming, requires extensive training Sensitive to stress, anxiety, light/noise conditions Low throughput	Excellent for fine motor analysis and chronic deficits Video-tracking increases precision	[58,117,157,158]
Rotarod Test	Automated and high-throughput Assesses coordination, endurance, fatigue resistance Highly reproducible when standardized	Animals may cling instead of walking Early after injury many cannot perform the task Device variability complicates cross-study comparisons	Useful for late-phase regeneration and motor learning Translational analogue: balance/endurance tests in humans	[61,116,157,159]
Von Frey Test (Mechanical Sensitivity)	Gold standard for mechanical allodynia Sensitive to changes in sensory recovery Quantitative threshold detection	Requires acclimation and careful handling Potential operator bias Stress alters withdrawal thresholds	Reflects clinically relevant mechanical hypersensitivity Useful across all phases of nerve regeneration	[64,120,160]
Hot Plate Test (Thermal Sensitivity)	Simple and widely used for thermal nociception Detects early sensory deficits and neuropathic pain	Affected by stress, anxiety, repeated testing Less specific for large-fiber injury	Monitors painful dysesthesia and sensory restoration	[64,121,122]
Pinch/Toe-Spread Reflex	Fast bedside-like test Detects early return of motor/sensory function	Qualitative, subjective Low sensitivity and poor resolution	Useful for screening before more complex tests	[161,162]
Gait Kinematics (2D/3D Motion Capture)	High-resolution analysis of joint angles, stride, limb trajectories Detects deficits not captured by SFI	Requires specialized equipment and software High cost	High translational relevance (clinical gait labs) AI-assisted analysis increases precision	[163,164]
Grip Strength Test	Measures functional muscle recovery Quantitative and simple	Influenced by motivation, stress Low specificity for sciatic nerve regeneration	Useful for assessing distal muscle reinnervation	[165,166]
Electrophysiology (NCV, CMAP, EMG)	Gold standard for conduction, remyelination, axonal continuity Highly sensitive to physiological recovery	Requires anesthesia and equipment Not a behavioral readout	Essential mechanistic correlate for functional tests Directly analogous to clinical nerve conduction studies	[67,167]
Automated Gait Analysis (CatWalk, DigiGait)	Precise paw placement, pressure, stride length Eliminates human variability Sensitive to subtle changes	Expensive systems Requires training and habituation	High translational relevance Suitable for early–late phase recovery monitoring	[157,163,168]
High-Resolution Ultrasound	Non-invasive, real-time assessment of peripheral nerve integrity Visualizes intraneural architecture Detects nerve swelling, discontinuity, fibrosis, and neuroma formation	Operator-dependent, requires expertise Limited penetration depth for deep nerves Reduced resolution compared to histology	Widely used clinically for peripheral nerve evaluation Enables longitudinal in vivo monitoring Strong histological–sonographic correlation Bridges preclinical and clinical nerve assessment	[71,72,73]
Optical Projection Tomography (OPT)	High-resolution 3D visualization of nerve architecture ex vivo Enables volumetric analysis of fascicles and branching patterns Preserves spatial relationships between nerve components	Ex vivo technique only Requires tissue clearing and labeling Limited availability and throughput	Excellent reference standard for validating in vivo imaging Useful for detailed structural analysis in preclinical studies Complements ultrasound and histology	[74,169,170]
Magnetic Resonance Microscopy (MRM)	High-resolution imaging of nerve microstructure Non-destructive visualization of fascicular organization Sensitive to myelin and axonal integrity	Mostly limited to ex vivo or small-animal imaging High cost and limited accessibility Long acquisition times	Provides multiscale structural reference data Useful for correlating microstructure with functional outcomes	[74,171]
Advanced Magnetic Resonance Imaging (MRI) Techniques	Non-invasive in vivo evaluation of peripheral nerve integrity and regeneration Provides multiscale structural information on nerve continuity, edema, and fiber organization Diffusion-based metrics reflect axonal orientation and myelination	Lower spatial resolution than ultrasound or histology High cost and limited availability Motion sensitivity and complex post-processing	Already used clinically for peripheral nerve assessment High translational relevance Complements ultrasound by providing functional–structural metrics	[70,75,76]

## Data Availability

No new data were created or analyzed in this study. Data sharing is not applicable to this article.

## Abbreviations

The following abbreviations are used in this manuscript:

A2A	Adenosine A2
AI	Artificial Intelligence
ALA	Alpha-Lipoic Acid
Arg1	Arginase-1
BDNF	Brain-Derived Neurotrophic Factor
BNB	Blood–Nerve Barrier
cAMP	Cyclic Adenosine Monophosphate
CMAP	Compound Muscle Action Potential
CRMP-2	Collapsin Response Mediator Protein-2
dECM	Decellularized Extracellular Matrix
ECM	Extracellular Matrix
EMG	Electromyography
ERK	Extracellular Signal-Regulated Kinase
ES	Electrical Stimulation
GDNF	Glial Cell Line-Derived Neurotrophic Factor
HO-1	Heme Oxygenase-1
IL-6	Interleukin-6
LIF	Leukemia Inhibitory Factor
MAPK	Mitogen-Activated Protein Kinase
MBP	Myelin Basic Protein
MCP-1	Macrophage Chemoattractant Protein-1
MES	Mechano-Electrical Stimulation
ML	Machine Learning
mPTP	Mitochondrial Permeability Transition Pore
MRI	Magnetic Resonance Imaging
MRM	Magnetic Resonance Microscopy
Mst3b	Mammalian Sterile 20-like Kinase-3b
NCV	Nerve Conduction Velocity
ND-SENS	Neurodevelopment-inspired Self-Evolving Neural Scaffold
NFH	neurofilament high
NGF	Nerve Growth Factor
NMNAT2	Nicotinamide Mononucleotide Adenyltransferase 2
NT3	Neurotrophin 3
OPT	Optical Projection Tomography
P0	Protein Zero
PDGF	Platelet-Derived Growth Factor
PEI	Polyethylenimine
PEG	Polyethylene Glycol
PI3K/AKT	Phosphoinositide 3-Kinase/Protein Kinase B
PLA	Polylactic Acid
PLC-γ	Phospholipase C gamma
PLCL	Poly l-Lactic Acid-co-ε-Caprolactone
PLLA	Poly l-Lactic Acid
PMP22	Peripheral Myelin Protein 22
PRP	Platelet-Rich Plasma
PSN	Peripheral Nervous System
PVDF	Poly Vinylidene Fluoride
SAA	Serum Amyloid A
SARM1	Sterile Alpha and Armadillo Motif 1
SFI	Sciatic Functional Index
TGF-β	Transforming Growth Factor-β
TNF	Tumor Necrosis Factor
TrFE	Trifluoroethylene
VEGF	Vascular Endothelial Growth Factor
